# Unsupervised Feature Learning With Winner-Takes-All Based STDP

**DOI:** 10.3389/fncom.2018.00024

**Published:** 2018-04-05

**Authors:** Paul Ferré, Franck Mamalet, Simon J. Thorpe

**Affiliations:** ^1^Centre National de la Recherche Scientifique, UMR-5549, Toulouse, France; ^2^Brainchip SAS, Balma, France

**Keywords:** Spike-Timing-Dependent-Pasticity, neural network, unsupervised learning, winner-takes-all, vision

## Abstract

We present a novel strategy for unsupervised feature learning in image applications inspired by the Spike-Timing-Dependent-Plasticity (STDP) biological learning rule. We show equivalence between rank order coding Leaky-Integrate-and-Fire neurons and ReLU artificial neurons when applied to non-temporal data. We apply this to images using rank-order coding, which allows us to perform a full network simulation with a single feed-forward pass using GPU hardware. Next we introduce a binary STDP learning rule compatible with training on batches of images. Two mechanisms to stabilize the training are also presented : a Winner-Takes-All (WTA) framework which selects the most relevant patches to learn from along the spatial dimensions, and a simple feature-wise normalization as homeostatic process. This learning process allows us to train multi-layer architectures of convolutional sparse features. We apply our method to extract features from the MNIST, ETH80, CIFAR-10, and STL-10 datasets and show that these features are relevant for classification. We finally compare these results with several other state of the art unsupervised learning methods.

## 1. Introduction

Unsupervised pre-training methods help to overcome difficulties encountered with current neural network based supervised algorithms. Such difficulties include : the requirement for a large amount of labeled data, vanishing gradients during back-propagation and the hyper-parameters tuning phase. Unsupervised feature learning may be used to provide initialized weights to the final supervised network, often more relevant than random ones (Bengio et al., 2007). Using pre-trained weights tends to speed up network convergence, and may also increase slightly the overall classification performance of the supervised network, especially when the amount of labeled examples is small (Rasmus et al., 2015).

Unsupervised learning methods have recently regained interest due to new methods such as Generative Adverserial Networks (Goodfellow et al., 2014; Salimans et al., 2016), Ladder networks (Rasmus et al., 2015), and Variational Autoencoders (Kingma and Welling, 2013). These methods reach state of the art performances, either using top layer features as inputs for a classifier or within a semi-supervised learning framework. As they rely on gradient descent methods to learn the representations for their respective tasks, computations are done with 32-bits floating point values. Even with dedicated hardware such as GPUs and the use of 16-bits half-floats type (Gupta et al., 2015), floating point arithmetic remains time and power consuming for large datasets. Several works are addressing this problem by reducing the resolution of weights, activations and gradients during inference and learning phases (Stromatias et al., 2015; Esser et al., 2016; Deng et al., 2017) and have shown small to zero loss of accuracy with such supervised methods. Nevertheless, learning features both with unsupervised methods and lower precision remains a challenge.

On the other hand, Spiking Neural Networks (SNNs) propagate information between neurons using spikes, which can be encoded as binary values. Moreover, SNNs often use an unsupervised Hebbian learning scheme, Spike-Timing-Dependent-Plasticity (STDP), to capture representations from data. STDP uses differences of spikes times between pre and post-synaptic neurons to update the synaptic weights. This learning rule is able to capture repetitive patterns in the temporal input data (Masquelier and Thorpe, 2007). SNNs with STDP may only require fully feed-forward propagation to learn, making them good candidates to perform learning faster than backpropagation methods.

Our contribution is three-fold. First, we demonstrate that Leaky Integrate and Fire neurons act as artificial neurons (perceptrons) for temporally-static data such as images. This allows the model to infer temporal information while none were given as input. Secondly, we develop a winner-takes-all (WTA) framework which ensure a balanced competition between our excitatory neuron population. Third, we develop a computationally-efficient and nearly parameter-less STDP learning rule for temporally static-data with binary weight updates.

## 2. Related work

### 2.1. Spiking neural networks

#### 2.1.1. Leaky-integrate-and-fire model

Spiking neural networks are widely used in the neuroscience community to build biologically plausible models of neuron populations in the brain. These models have been designed to reproduce information propagation and temporal dynamics observable in cortical layers. As many models exists, from the most simple to the most realistic, we will focus on the Leaky-Integrate-and-Fire model (LIF), a simple and fast model of a spiking neuron.

LIF neurons are asynchronous units receiving input signals called spikes from pre-synaptic cells. Each spike *x*_*i*_ is modulated by the weight *w*_*i*_ of the corresponding synapse and added to the membrane potential *u*. In a synchronous formalism, at each time step, the update of the membrane potential at time *t* can be expressed as follow:

(1) $$\begin{array}{l} T\frac{\delta u\left(t\right)}{\delta t}=-\left(u\left(t\right)-u_{res}\right)+\overset{n}{\underset{i=1}{\sum }}w_{i}x_{i,t} \end{array}$$

Where T is the time constant of the neuron, *n* the number of afferent cells and *u*_*res*_ is the reset potential (which we also consider as the initial potential at *t*_0_ = 0).

When *u* reaches a certain threshold *T*, the neuron emits a spike to its axons and resets its potential to its initial value *u*_*res*_.

This type of network has proven to be energy-efficient Gamrat et al. (2015) on analog devices due to its asynchronous and sparse characteristics. Even on digital synchronous devices, spikes can be encoded as binary variables, therefore carrying maximum information over the minimum memory unit.

#### 2.1.2. Rank order coding network

A model which fits the criteria of processing speed and adaptation to images data is the rank order coding SNN (Thorpe et al., 2001). This type of network processes the information with single-step feed-forward information propagation by means of the spike latencies. One strong hypothesis for this type of network is the possibility to compute information with only one spike per neuron, which has been demonstrated in rapid visual categorization tasks (Thorpe et al., 1996). Implementations of such networks have proven to be efficient for simple categorization tasks like frontal-face detection on images (Van Rullen et al., 1998; Delorme and Thorpe, 2001).

The visual-detection software engine SpikeNet Thorpe et al. (2004) is based on rank order coding networks and is used in industrial applications including face processing for interior security, intrusion detection in airports and casino games monitoring. Also, it is able to learn new objects with a single image, encoding objects with only the first firing spikes.

The rank order model SpikeNet is based on a several layers architecture of LIF neurons, all sharing the time constant T, the reset potential *u*_*res*_ and the spiking threshold *T*. During learning, only the first time of spike of each neuron is used to learn a new object. During inference, the network only needs to know if a neuron has spiked or not, hence allowing the use of a binary representation.

### 2.2. Learning with spiking neural networks

#### 2.2.1. Deep neural networks conversion

The computational advantages of SNNs led some researchers to convert fully learned deep neural networks into SNNs (Diehl et al., 2015, 2016), in order to give SNNs the inference performance of back-propagation trained neural networks.

However, deep neural networks use the back-propagation algorithm to learn the parameters, which remains a computationally heavy algorithm, and requires enormous amounts of labeled data. Also, while some researches hypothesize that the brain could implement back-propagation (Bengio et al., 2015), the biological structures which could support such error transmission process remain to be discovered. Finally, unsupervised learning within DNNs remains a challenge, whereas the brain may learn most of its representations through unsupervised learning (Turk-Browne et al., 2009). Suffering from both its computational cost and its lack of biological plausibility, back-propagation may not be the best learning algorithm to take advantage of SNNs capabilities.

On the other hand, researches in neuroscience have developed models of unsupervised learning in the brain based on SNNs. One of the most popular model is the STDP.

#### 2.2.2. Spike timing dependent plasticity

Spike-Timing-Dependent-Plasticity is a biological learning rule which uses the spike timing of pre and post-synaptic neurons to update the values of the synapses. This learning rule is said to be Hebbian (“What fires together wires together”). Synaptic weights between two neurons updated as a function of the timing difference between a pair or a triplet of pre and post-synaptic spikes. Long-Term Potentiation (LTP) or a Long-Term Depression (LTD) are triggered depending on whether a presynaptic spike occurs before or after a post-synaptic spike, respectively.

Formulated two decades ago by Markram et al. (1997), STDP has gained interest in the neurocomputation community as it allows SNN to be used for unsupervised representation learning (Kempter et al., 2001; Rao and Sejnowski, 2001; Masquelier and Thorpe, 2007; Nessler et al., 2009). The features learnt in low-level layers have also been shown to be relevant for classification tasks combined with additional supervision processes in the top layers (Beyeler et al., 2013; Mozafari et al., 2017). As such STDP may be the main unsupervised learning mechanisms in biological neural networks, and shows nearly equivalent mathematical properties to machine learning approaches such as auto-encoders (Burbank, 2015) and non-negative matrix factorization (Carlson et al., 2013; Beyeler et al., in review).

We first consider the basic STDP pair-based rule from Kempter et al. (2001). Each time a post synaptic neuron spikes, one computes the timing difference Δ*t* = *t*_*pre*_−*t*_*post*_ (relative to each presynaptic spike) and updates each synapse *w* as follows:

(2) $$\Delta w=\;\{\begin{array}{c} A^{+}.e^{\frac{\Delta t}{{Τ}_{+}}}\text{ if }\Delta t<0\\ A^{-}.e^{\frac{\Delta t}{{Τ}_{-}}}\text{ otherwise} \end{array}$$

where *A*_+_ > 0, *A*_−_ < 0, and $T_{+},T_{-}>0$. The top and bottom terms in this equation are respectively the LTP and LTD terms.

This update rule can be made highly computationally efficient by removing the exponential terms $e^{\frac{\Delta t}{T}}$, resulting in a simple linear time-dependent update rule.

Parameters *A*_+_ and *A*_−_ must be tuned on order to regularize weight updates during the learning process. However in practice, tuning these parameters is a tedious task. In order to avoid weight divergences, networks trained with STDP learning rule should also implement stability processes such as refractory periods, homoeostasis with weight normalization or inhibition. Weight regularization may also be implemented directly by reformulating the learning rule equations. For instance in Masquelier and Thorpe (2007), the exponential term in Equation (2) is replaced by a process which guaranties that the weights remain in the range [0…1] :

(3) $$\Delta w=\;\{\begin{array}{c} A_{+}.w.(1-w)\text{ if }\Delta t<0\\ A_{-}.w.(1-w)\text{ otherwise} \end{array}$$

Note that in Equation (3), the amplitude of the update is independent from the absolute time difference between pre and post-synaptic spikes, which only works if pairs of spikes belongs to the same finite time window. In Masquelier and Thorpe (2007) this is guaranteed by the whole propagation schemes, which is applied on image data and rely on a single feedforward propagation step taking into account only one spike per neuron. Thus the maximum time difference between pre and post-synaptic spikes is bounded in this case.

### 2.3. Regulation mechanisms in neural networks

#### 2.3.1. WTA as sparsity constrain in deep neural networks

Winner-takes-all (WTA) mechanisms are an interesting property of biological neural networks which allow a fast analysis of objects in exploration tasks. Following de Almeida et al. (2009), gamma inhibitory oscillations perform a WTA mechanism independent from the absolute activation level. They may select the principle neurons firing during a stimulation, thus allowing, e.g., the tuning of narrow orientation filters in V1.

WTA has been used in deep neural networks in Makhzani and Frey (2015) as a sparsity constraint in autoencoders. Instead of using noise or specific loss functions in order to impose activity sparsity in autoencoder methods, the authors propose an activity-driven regularization technique based on a WTA operator, as defined by Equation (4).

(4) $$WTA(X,d)=\{\begin{array}{c} X_{j}\text{ if}\left|X_{j}\right|=\underset{k\in d}{max}\left(\left|X_{k}\right|\right)\\ 0\text{ otherwise} \end{array}$$

where *X* is a multidimensional matrix and *d* is a set of given dimensions of *X*.

After definition of a convolutional architecture, each layer is trained in a greedy layer-wise manner with representation from the previous layer as input. To train a convolutional layer, a WTA layer and a deconvolution layer are placed on top of it. The WTA layer applies the WTA operator on the spatial dimensions of the convolutional output batch and retains only the *n*_*p*_% first activities of each neuron. This way for a given layer with *N* representations map per batch and *C* output channels, only *N*.*n*_*p*_.*C* activities are kept at their initial values, all the others activation values being zeroed. Then the deconvolutional layer attempts to reconstruct the input batch.

While this method demonstrates the potential usefulness of WTA mechanisms in neural networks, it still relies on computationally heavy backpropagation methods to update the weights of the network.

#### 2.3.2. Homosynaptic and heterosynaptic homeostasis

In their original formulation, Hebbian-type learning rule (STDP, Oja rule, BCM rule) does not have any regulation process. The absence of regulation in synaptic weights may impact negatively the way a network learns. Hebbian learning allows the synaptic weights to grow indefinitely, which can lead to abnormally high spiking activity and neurons to always win the competitions induced by inhibitory circuits.

To avoid such issues, two types of homeostasis have been formulated.

Homosynaptic homeostasis acts on a single synapse and is depends on its respective inputs and outputs activity only. This homeostatic process can be modeled with a self-regulatory term in the Hebbian rule as in Masquelier and Thorpe (2007) or as a synaptic scaling rule depending on the activity driven by the synapse as in Carlson et al. (2013).

Heterosynaptic homeostasis is a convenient way to regulate the synaptic strength of a network. The model of such homeostasis takes into account all the synapses connected to a given neuron, all the synapses in a layer (like the L2 loss weight decay in deep learning) or at the network scale. Biological plausibility of such process is still discussed. Nevertheless, some evidences of heterosynaptic homeostasis have been observed in the brain to compensate runaway dynamics of synaptic strength introduced by Hebbian learning (Royer and Paré, 2003; Chistiakova et al., 2014). It then plays an important role in the regulation of spiking activity in the brain and is complementary to homosynaptic plasticity.

### 2.4. Neural networks and image processing

Image processing with neural networks is performed with multiple layers of spatial operations (like convolutions, pooling, and non-linearities), giving the name Deep Convolutional Neural Networks to these methods. Their layer architecture is directly inspired from the biological processes of the visual cortex, in particular from the well known HMAX model (Riesenhuber and Poggio, 1999), except that the layers' weights are learnt with back-propagation. Deep CNN models use a single-step forward propagation to perform a given task. Even if convolutions on large maps may be computationally heavy, all the computations are done through only one pass in each layer. One remaining advantage of CNNs is their ability to learn from raw data, such as pixels for images or waveforms for audio.

On the other hand, since SNNs use spikes to transmit information to the upper layers, they need to perform neuron potential updates at each time step. Hence, applying such networks with a convolutional architecture requires heavy computations once for each time step. However, spikes and synaptic weights may be set to a very low bit-resolution (down to 1 bit) to reduce this computational cost Thorpe et al. (2004). Also, STDP is known to learn new representations with a few iterations Masquelier et al. (2009), theoretically reducing the number of epochs required to converge.

## 3. Contribution

Our goal here is to apply STDP in a single-step feed-forward formalism directly from raw data, which should be beneficial in the cases where training times and data labeling are issues. Thus we may select a neural model which combines the advantages of each formalism in order to reduce the computational cost during both training and inference.

### 3.1. Feedforward network architecture

#### 3.1.1. Neural dynamics

Here, we will consider the neural dynamics of a spiking LIF network in presence of image data. Neural updates in the temporal domain in such neural architecture are as defined by Equation (1).

Since a single image is a static snapshot of visual information, all the *x*_*i, t*_ are considered constant over time. Hence $\overset{n}{\underset{i=1}{\sum }}w_{i}.x_{i,t}$ is also constant over time under the assumption of static synaptic weights during the processing of the current image.

Let us define $v_{in}=\overset{n}{\underset{i=1}{\sum }}w_{i}.x_{i,t},\forall t$ the total input signal to the neuron. Let us also determine *u*(*t*_0_ = 0) = *u*_*res*_ as an initial condition. As *v*_*in*_ is constant over time, we can solve the differential equation of the LIF neuron, which gives:

(5) $$\begin{array}{l} \begin{array}{c} T\frac{\delta u\left(t\right)}{\delta t}=-\left(u\left(t\right)-u_{res}\right)+v_{in}\\ \Rightarrow \;u\left(t\right)=-v_{in}.e^{\frac{-t}{T}}+u_{res}+v_{in}\;\forall t>0 \end{array} \end{array}$$

The precise first spike-time of a neuron given its spiking threshold *T* is given by :

(6) $$\begin{array}{l} t_{s}=-T.log\left(1+\frac{u_{res}-T}{v_{in}}\right) \end{array}$$

Since Equation (6) decreases monotonically wrt. *v*_*in*_, we can recover the intensity-latency equivalence. The relative order of spike-times is also known since *v*_*in*, 1_ > *v*_*in*, 2_ → *t*_*s*, 1_ < *t*_*s*, 2_.

#### 3.1.2. Equivalence with artificial neuron with ReLU activation

Thus from Equation (6), for each neuron we can determine the existence of a first spike, along with its precise timing. Hence, since we are only concerned with the relative times of first spikes across neurons, one can replace the computation at each time-step by a single-step forward propagation given the input intensity of each neuron.

The single-step forward propagation correspond to LIF integration when *t* → ∞. As we are first looking for the existence of any *t*_*s*_ such that *u*(*t*_*s*_) > *T*:

(7) $$\begin{array}{l} \underset{t\to \infty }{\lim}u(t)-T=\underset{t\to \infty }{\lim}-v_{in}.e^{\frac{-t_{s}}{Τ}}+u_{res}+v_{in}-T\\ \;=u_{res}+v_{in}-T \end{array}$$

Having $\;v_{in}=\overset{n}{\underset{i=1}{\sum }}w_{i}.x_{i}\;$ and *b* = *u*_*res*_ − *T*,

(8) $$\begin{array}{l} \underset{t\to \infty }{\lim}u\left(t\right)-T=b+\overset{n}{\underset{i=1}{\sum }}w_{i}.x_{i} \end{array}$$

which is the basic expression of the weighted sum of a perceptron with bias.Also, *t*_*s*_ exists if and only if $b+\overset{n}{\underset{i=1}{\sum }}w_{i}.x_{i}>0$, which shows the equivalence between LIF neurons with constant input at infinity and the artificial neuron with rectifier activation function (ReLU).

This demonstration can be generalized to local receptive fields with weight sharing, and thus we propose to replace the time-step computation of LIF neurons, by common GPU optimized routines of deep learning such as 2D convolutions and ReLU non-linearity. This allows us to obtain in a single-step all the first times of spikes -inversely ordered by their activation level- and nullified if no spike would be emitted in an infinite time. Moreover, these different operations are compatible with mini-batch learning. Hence, our model is also capable of processing several images in parallel, which is an uncommon feature in STDP-based networks.

#### 3.1.3. Winner-takes-all mechanisms

Following the biological evidence of the existence of WTA mechanisms in visual search tasks (de Almeida et al., 2009) and the code sparsity learned with such processes (Makhzani and Frey, 2015), we may take advantage of WTA to match the most repetitive patterns in a given set of images. Also, having to learn only these selected regions should drastically decrease the number of computations required for the learning phase (compared to dense approaches in deep learning and SNN simulations). Inspired by this biological mechanism, we propose to use three WTA steps as sparsifying layers in our convolutional SNN architecture.

The first WTA step is performed on feature neighborhood with a max-pooling layer on the convolution matrix with kernel size *k*_*pool*_ > = *k*_*conv*_ and stride *s*_*pool*_ = *k*_*conv*_. This acts as a lateral inhibition, avoiding the selection of two spikes from different kernels in the same region.

Next we perform a WTA step with the WTA operation (Equation 4) on the channel axis for each image (keeping at each pooled pixel, the neuron that spikes first). This forces each kernel to learn from different input patches.

The third WTA step is performed with WTA operation on spatial axes as in Makhzani and Frey (2015). This forces the neuron to learn from the most correlated patch value in the input image.

The WTA operation (Equation 4) is not to be confused with the Maxout operation from Goodfellow et al. (2013) and the max pooling operation, since these latter squeeze the dimensions on which they are applied, while the WTA operation preserves them.

Then we extract the indexes of the selected outputs along with their sign and their corresponding input patch. Extracted input patches are organized in *k* subsets, each subset corresponding to one output channel. These matrices will be refered to as follow :

- *Y*_*k*_ : matrices of selected outputs, of dimension (*m*_*k*_, *c*_*out*_)
- *X*_*k*_ : matrices of selected patches, of dimension (*m*_*k*_, *c*_*in*_ × *h*_*in*_ × *w*_*in*_)
- *W* : matrices of filters, of dimension (*c*_*in*_ × *h*_*in*_ × *w*_*in*_, *c*_*out*_)

with *m*_*k*_ the number of selected indexes and patches for neuron *k* ∈ [1…*c*_*out*_], *c*_*o*_*ut* the number of channels (or neurons) of the output layer, and *c*_*i*_*n, h*_*i*_*n, w*_*i*_*n* are the receptive field size (resp. channel, height and width). Note that at most one output is selected per channel and per image, *m*_*k*_ ≤ *N*.

The WTA in our model has two main advantages. First, it allows the network to learn faster on only a few regions of the input image. Second, classical learning frameworks use the mean of weights gradient matrix to update the synaptic parameters. By limiting the influence of averaging on the gradient matrix, synaptic weights are updated according to the most extreme values of the input, which allow the network to learn sparse features.

**Figure 1:**
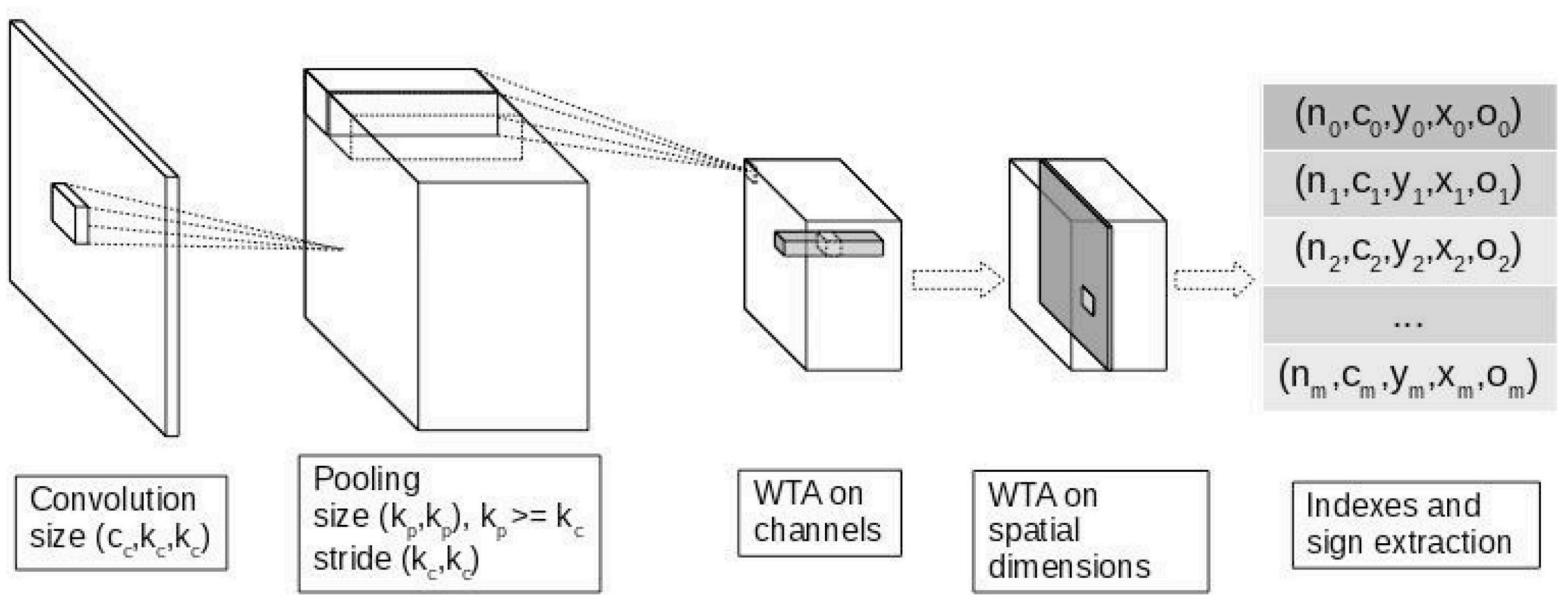
Processing chain for the region WTA.

Note that the network is able to propagate relative temporal information through multiple layer, even though presented inputs lack this type of data. It is also able to extract regions which are relevant to learn in terms of information maximization. The full processing chain for propagation and WTA is shown in Figure 1.

### 3.2. Binary hebbian learning

#### 3.2.1. Simplifying the STDP rule

Taking inspiration from the STDP learning rule, we propose a Hebbian correlation rule which follows the relative activations of input and output vectors.

Considering the input patch value *x*_*n, i*_ ∈ *X*_*n*_, *n* ∈ [1…*m*_*k*_], *i* ∈ [1…*c*_*in*_ × *h*_*in*_ × *w*_*in*_], the corresponding weight value *w*_*k, i*_, the selected output value *y*_*k*_ ∈ *Y*_*k*_ and a heuristically defined threshold *T*_*l*_, the learning rule is described in Equation (9).

(9) $$\Delta w_{k,i}=\{\begin{array}{l} sign(x_{n,i}).sign(y_{k})\;\text{if}\;|x_{n,i}|\;>T_{l}\\ -sign(w_{k,i})\;\text{otherwise} \end{array}$$

The learning rule is effectively Hebbian as shown in the next paragraph and can be implemented with lightweight operations such as thresholding and bit-wise arithmetic.

Also, considering our starting hypotheses, where we limit to one the number of spikes per neuron during a full propagation phase for each image, it is guaranteed that, for any pair of pre and post-synaptic neuron, the choice of LTP or LTD exist and is unique for each image presentation. These hypotheses are similar to the ones in Masquelier and Thorpe (2007), where these conditions simulates a single wave of spikes within a range of 30 ms.

#### 3.2.2. Equivalence to hebbian learning in spiking networks

In this section we show the Hebbian behavior of this learning rule. For this, we first focus on the “all positive case” (*x, y, w* ∈ *R*+) and will explain in the next section the extension to symmetrical neurons.

In the case of “all positive,” the Equation (9) can be rewritten as Equation (10).

(10) $$\Delta w_{k,i}=\{\begin{array}{l} 1\;\text{if}\;x_{k,i}>u(t_{post})\\ -1\;\text{otherwise} \end{array}$$

This rule tends to increase the weights when the input activity is greater than a threshold (here the post-synaptic neuron firing threshold), and decreases it otherwise.

Equation (10) is equivalent to the pair-based STDP rule presented in Equation (2) removing the exponential term and using *A*_+_ = 1 and *A*_−_ = −1.

#### 3.2.3. Extension to symmetric neurons

We have demonstrated that the proposed learning rule is effectively Hebbian in the case where *x, w, y* ∈ ℝ_+_. Our learning rule also takes into account negative values of *x, w, y*. In biological networks models, negative values do not seem to make much sense since firing rates and synaptic conductance are expressed in units defined only in ℝ_+_.

Nevertheless, negative values are used in many spiking networks models in the very first layer of visual features. For instance, ON-centered-OFF-surround and OFF-centered-ON-surround filters (also known as *Mexican hat* filters) are often used to pre-process an image in order to simulate retinal cells extracting gradients. These two filters are symmetric with respect to the origin. Hence a common computational optimization is to apply only one of the two filters over the image, separating negative and positive resulting values as OFF and ON activities, respectively.

We extend this computational trick to neurons in any neural layer under the hypothesis that negative values for *x, w, y* corresponds to activities and weights of synaptically symmetric neurons. For a neuron with constant input activity *X* and synaptic weights *W* of size *n*, we can express its output activity $y=\overset{n}{\underset{i=1}{\sum }}X_{i}\times W_{i}$. If *y* < 0, we can convert it to a positive value using the synaptically opposite weights $\overset{n}{\underset{i=1}{\sum }}X_{i}\times -W_{i}=-y$.

Under the hypothesis of the existence of a pair-wise competition between neurons with symmetric weights (for instance with inhibition), this computational trick remains biologically plausible.

Considering now the proposed learning rule, the weights update given *x, y*, and *w* is shown in Table 1. In this table, the first spikes (|*x*| > *T*) will induce an update of the weight to increase the |*y*| (Δ*w* = *sign*(*y*).*sign*(*x*)). Meanwhile, the weights corresponding to the last spike will be reduced (Δ*w* = −*sign*(*w*)).

**Table 1:** Weight update given x, y, and w following the proposed learning rule (Equation 9).

	***x* < −*T***	**−*T*<*x*<*T***	***x*>*T***
*y*>0	−1	−*sign*(*w*)	+1
*y* < 0	+1	−*sign*(*w*)	−1

With this framework the choice of the parameter *T*_*l*_ is critical. Thanks to the WTA mechanism developed, the selection of a neuron for learning is performed disregarding its firing threshold *T*, set to zero in practice. Hence contrary to Masquelier and Thorpe (2007), we cannot rely on the precise firing threshold of the neuron. In order to approximate this threshold, we developed two strategies described in the next paragraphs. These strategies are made adaptative such that the learning rule can be invariant to contrast variation. Also the adaptative behavior of this threshold avoids to tune an additional parameter in the model.

#### 3.2.4. Hard percentile threshold

The first strategy applied follows the STDP learning rule, which fixes a time constant for LTP and LTD. In our framework this is implemented as a percentile of the input activity to map their influence in the spike. For each input vector *x*_*n*_ ∈ *X*_*k*_ ∀ *k*, we compute the patch threshold *T*_*l*_ as the minimum value in the local *p*_*n%*_ percentile. *p*_*n%*_ is manually set and global for all the patches.

(11) $$\Delta w_{k,i}=\{\begin{array}{l} -sign(w_{k,i})\text{ if }|x_{n,i}|\leq p_{n\%}\\ sign(x_{n,i}).sign(y_{k})\text{ otherwise} \end{array}$$

However, we have seen experimentally that the threshold tuning may be cumbersome. As it regulates the sparsity of the synaptic weight matrix, fixing the sparsity manually may lead to unsatisfying results. Also, getting the percentiles uses the index-sorting operation which is time consuming.

#### 3.2.5. Average correlation threshold

We propose a second strategy which relies on the computation of an adaptative threshold between LTP and LTD. For each input vector *x*_*n*_ ∈ *X*_*k*_ ∀ *k* we compute the sign correlated input activation as $\hat{x_{n,i}}=x_{n,i}.sign\left(w_{k}\right).sign\left(y_{k}\right)$. Next we compute the threshold *T*_*l*_ as the mean of $\hat{x_{n}}$. Then we apply the learning rule in Equation (9).

With this strategy, the learning rule is also equivalent to Equation (12), which is straightforward to implement since it avoids conditional branching.

(12) $$\begin{array}{l} \Delta w_{k,i}=sign\left(x_{n,i}.sign\left(y_{k}\right).sign\left(w_{k,i}\right)-T_{l}\right).sign\left(w_{k,i}\right) \end{array}$$

Using the mean sign corrected input activation as a threshold, the model is able to be invariant to local contrasts. It also requires the calculation of the mean and a thresholding, two operations that are much faster than sorting. Finally, the adaptative behavior of such a threshold automate the sparsity of synaptic weights.

#### 3.2.6. Computing updates from a batch of images

Since our method allows the propagation of several images at the same time through mini-batch, we can also adapt our learning rule when batches of images are presented. Since biological visual systems never deal with batches of dozen images at once, the following proposal is a computational trick to accelerate the learning times, not a model of any existing biological feature.

When all the update vectors have been computed, the weight update vector for the current batch is obtained through the binarization of the sum of all the update vector for the corresponding kernel. We finally modulate the update vector with a learning rate λ.

(13) $$\begin{array}{l} U_{n,i}=\overset{m_{k}}{\underset{k=1}{\sum }}\Delta w_{k,i} \end{array}$$

(14) $$\Delta W_{k,i}=\{\begin{array}{c} -1\text{ if }U_{n,i}\leq 0\\ 1\text{ otherwise} \end{array}$$

(15) $$\begin{array}{l} W_{k,i}=W_{k,i}+\lambda .\Delta W_{k,i} \end{array}$$

#### 3.2.7. Weight normalization through simple statistics

Since each update step adds +λ or −λ to the weights, a regularization mechanism is required to avoid the weights growing indefinitely. Also we want to maintain a fair competition between neurons of the same layer, thus the total energy of the weights should be the same for all the neurons.

We propose a simple model of heterosynaptic homeostasis in order to regulate the weights of each neuron.We chose to normalize the weights of each neuron *k* by mean centering and standardization by variance. Hence, after each update phase, the normalization is done as follows :

(16) $$\begin{array}{l} W_{k}=\frac{W_{k}-\mu \left(W_{k}\right)}{\sigma^{2}\left(W_{k}\right)} \end{array}$$

This way, even neurons which did not learn a lot during the previous epochs can win a competition against the others. In practice, we set λ in an order of magnitude of 10^−1^ and halved it after each epoch. Given the order of magnitude of λ and the unit variance of *W*_*k*_, we know that ninety-five percent of the weights belongs to the interval [−1.5…1.5]. In fact, only a few batches of images are necessary to modify the influence of a given afferent. Two neurons responding to a similar pattern can thus diverge and specialize on different patterns in less than a dozen training batches.

As a detail, if the WTA region selected is small, some neurons may learn parts of patterns already learned by an other one. Since $\sigma^{2}\left(W_{k}\right)=1$ and most of the weights are equal to zero, the values of the remaining weights would grow very large. This can end up in multiple neurons learning almost identical patterns. We have observed that clipping weights after normalization between the range [−2…2] prevents this situation.

### 3.3. Multi-layer architectures with binary STDP

This proposed approach is able to learn a multi-layer convolutional architecture as defined by the user. It does not require a greedy layer-wise training, all the convolutional layers can be trained in parallel. We can optionally apply a non-linearity, a downsampling operation or a normalization after each convolution layer.

Once all the features layers have learned, the whole features architecture can process images as a classical convolutional neural network in order to obtain the new representations.

## 4. Experiments and results

### 4.1. Method

The proposed method learns, unsupervised, convolutional features from image data. In order to validate our approach, we evaluated the learnt features on four different classification datasets : MNIST, ETH80, CIFAR10, and STL10. Architectures and hyper-parameters were tuned separately for each dataset, details being given in the relevant sections.

The overall evaluation method remains the same for each dataset. The proposed framework will be used to learn one or several convolutional layer with the simplified STDP. In order to show the faster convergence of features with our method, we will only train these layer with a subset of the full training dataset with very few epochs.

Once the features are learnt, we show qualitatively the learnt features for each dataset. To quantitatively demonstrate their relevance, we use the extracted features as input to a supervised classifier. Although as state of the art classification are deep learning systems, we use a simple Multi-Layer Perceptron (MLP) with zero, one, or two hidden layers (depending on the dataset) taking as inputs the learnt features with the proposed solution.

For all the experiments, we started with a lightweight network architecture (the simplest available in the literature if available), and incrementally added complexity until further additions stopped improving performance. The classifier on top of the network starts as linear dense layer with as many neurons as the number of classes, and is complexified with intermediate layers as the architectural-tuning goes on.

We compare our results with other state of the art unsupervised feature learning methods specific for each dataset.

### 4.2. MNIST

The MNIST dataset contains 60,000 training images and 10,000 testing images of size 28 × 28 containing handwritten digits from 0 to 9. MNIST digits are written in white on a black background, hence pixel values are distributed across two modes. Considering the data distribution and the limited number of classes, MNIST may be considered as an easy classification task for current state-of-the-art methods. As a matter of fact, neural based methods do not need deep architectures in order to perform well on this dataset. Light-weight architectures can be defined in order to explore issues with the developed method. Once the method has satisfying results on MNIST, more complex datasets may be tackled.

**Figure 2:**
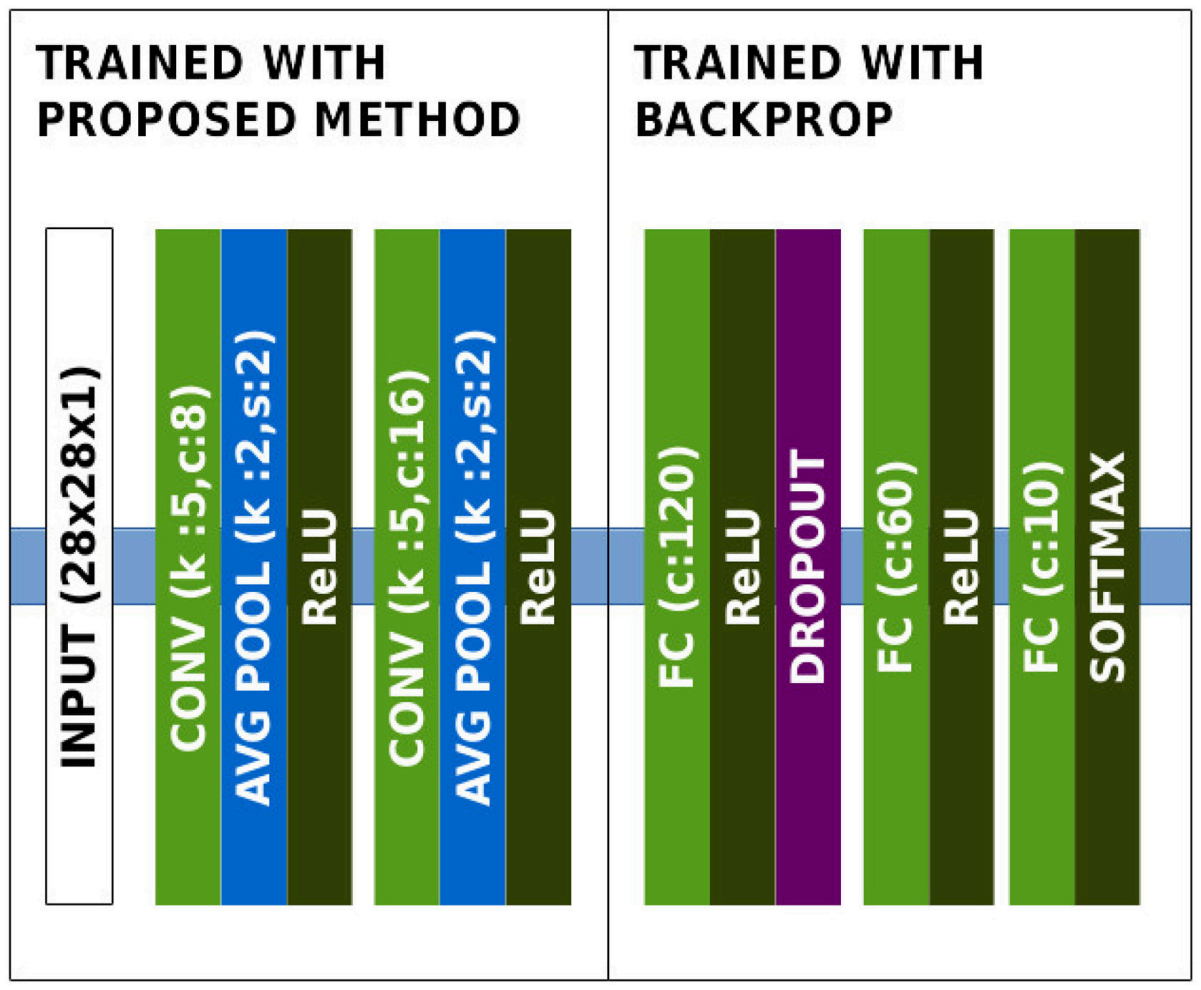
Architecture of the network in the MNIST experiment.

To perform classification on this dataset, we defined a lightweight convolutional architecture of features close to LeNet LeCun et al. (1998), presented in Figure 2. Since achieving high classification accuracy on MNIST is easy with a high number of neurons per layer, the number of neurons per layer was kept as low as possible in order to actually verify the relevance of the features.

**Figure 3:**
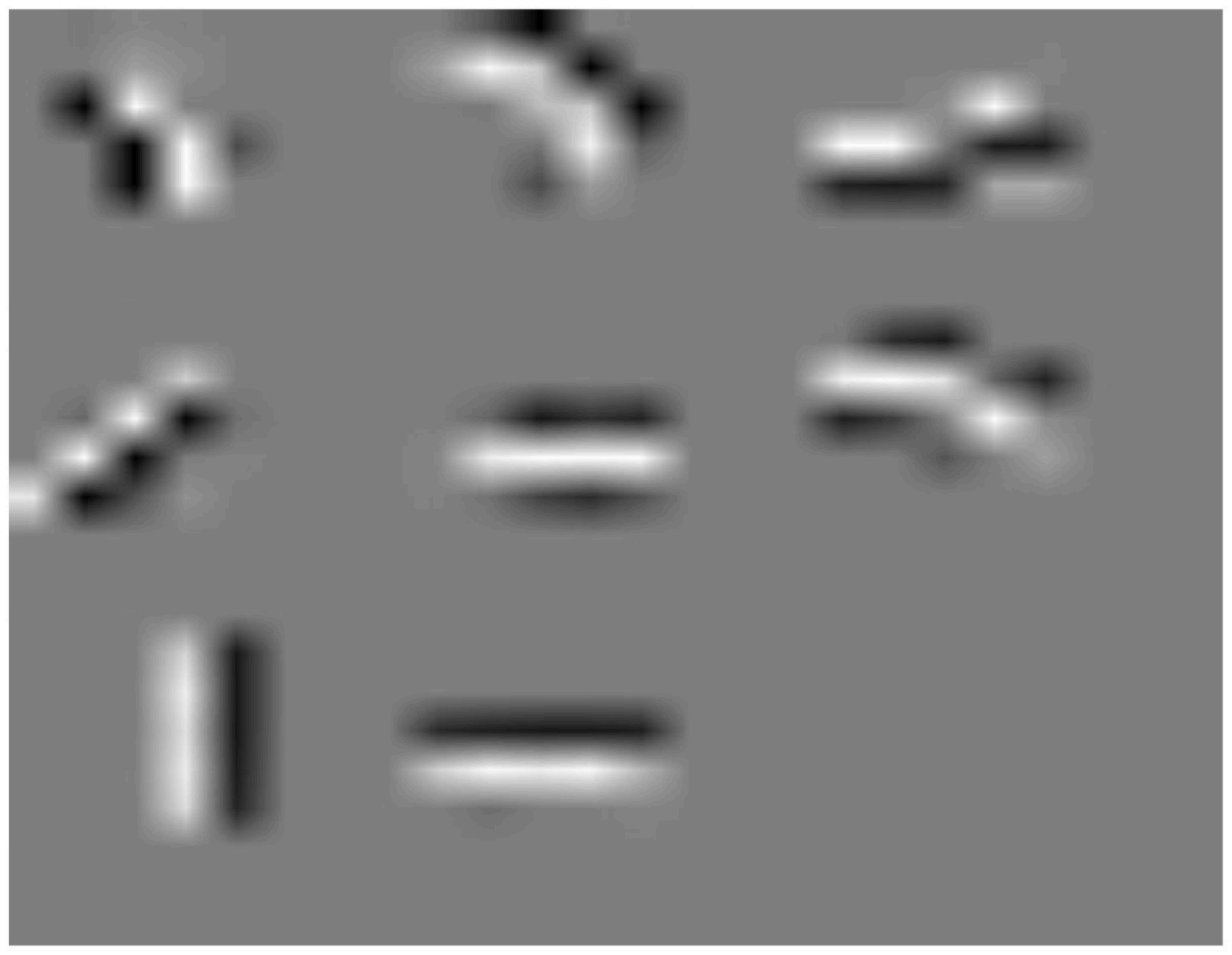
Eight 5 × 5 features learned from MNIST dataset on raw images.

Unsupervised learning was performed over only 5,000 random images from the dataset for 5 epochs, which only represents 25,000 image presentations. A visualization of the learnt features is shown in Figure 3.

Once the features were learnt, we used a two-hidden layers MLP to perform classification over the whole transformed training set. The learnt features and classifier were then run on all the testing set images in order to get the test error rate.

Classification performances are reported in Table 2. While the best methods in the state-of-the-art reach up to 99.77% accuracy, we did not report these results since these approaches use supervised learning with data augmentation, which is outwith the scope of this paper. All the reported results were obtained without data augmentation and using unsupervised feature learning.

**Table 2:** MNIST accuracy.

**Method**	**Accuracy (%)**
SDNN (Kheradpisheh et al., 2016)	98.40
Two layer SNN (Diehl and Cook, 2015)	95.00
PCA-Net (Chan et al., 2014)	98.94
Our method	98.49

Our approach performs as well as SDNN since they are structurally close, reaching state-of-the-art performance without fine-tuning and data-augmentation. While PCA-Net has better performance, learning was done on twice the number of samples we used. Doubling the number of samples to match the same number used for PCA-Net (10,000) did not improve the performance of our method.

### 4.3. ETH80

The ETH80 (Leibe and Schiele, 2003) contains 3,280 color images of eight different object categories (apple, car, cow, cup, dog, horse, pear, tomato). Each category contains 10 different object instances taken from 41 points of view. This dataset is interesting since the number of available images is limited and contains a lot of variability in 3D rotations. It allows us to evaluate the generalization potential of the features and their robustness to changes in viewpoint.

As the number of samples is restrained here, we performed both unsupervised and supervised learning on half the dataset (1,640 images chosen randomly). The other half was used as the test set.

**Figure 4:**
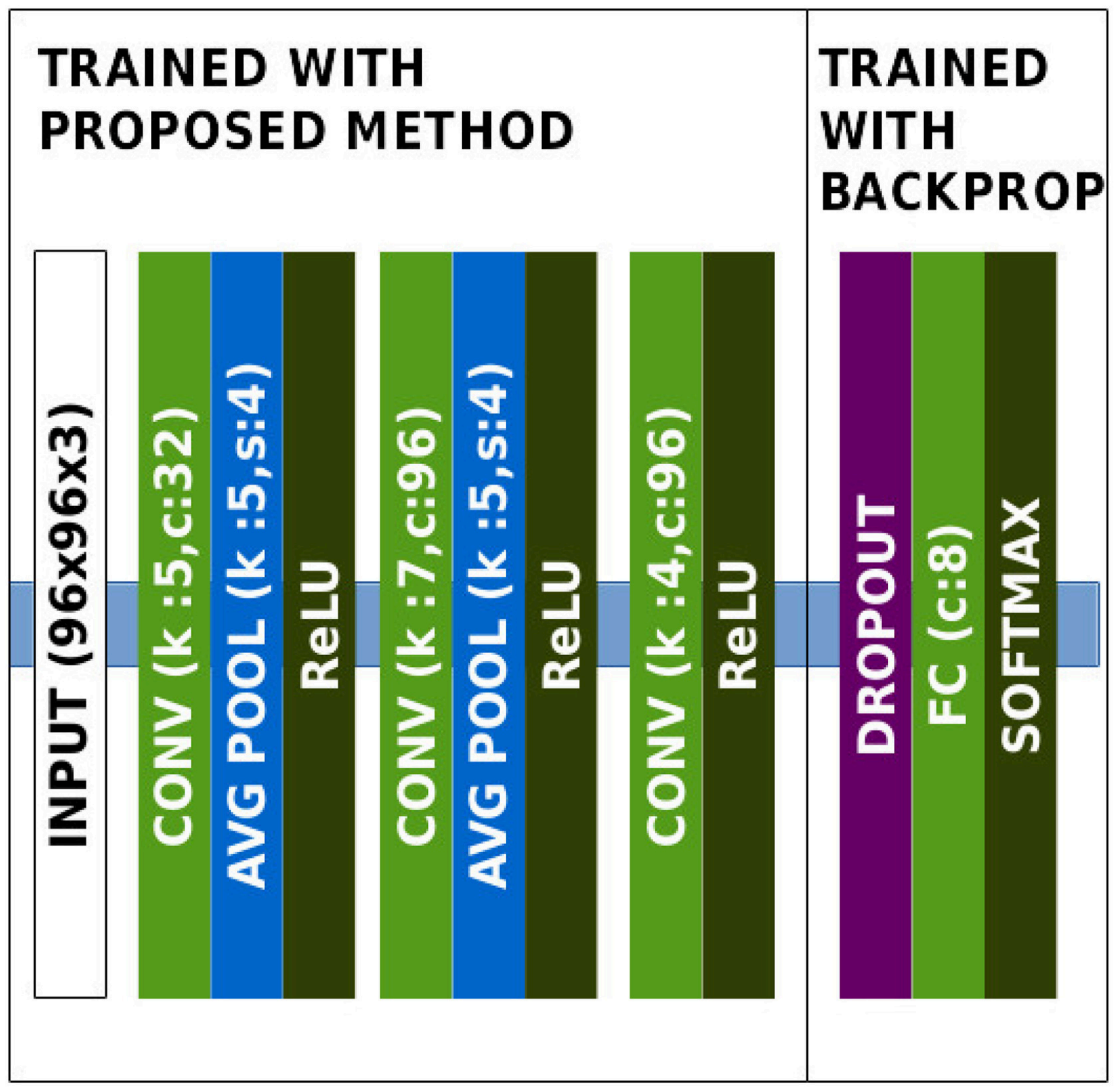
Architecture of the network in the ETH80 experiment.

We compare our approach to the classical HMAX model and to Kheradpisheh et al. (2016). The architectures for unsupervised and supervised part are shown in Figure 4. Learning visual features becomes more and more difficult with the proposed method as we add convolutional layers on top of the network. Since ETH80 images are large (96 × 96), we apply pooling with a stride of 4 in order to quickly reduce the dimensions over the hierarchy.

Results are reported in Table 3. While our approach does not reach the same performance as Kheradpisheh et al. (2016), it is able to learn features relevant for a classification task with multiple points of view of different objects.

**Table 3:** ETH80 results.

**Method**	**Accuracy (%)**
HMAX (Riesenhuber and Poggio, 1999)	69.0
SDNN (Kheradpisheh et al., 2016)	82.8
Our method	75.2

### 4.4. CIFAR-10

The CIFAR-10 dataset (Krizhevsky, 2009) is a dataset for classification of natural images from 10 classes (airplane, automobile, bird, cat, deer, dog, frog, horse, ship, and truck). The dataset is split into three with 60,000 training, 10,000 validation, and 10,000 testing images. Images are a subset of the 80 million tiny images dataset (Torralba et al., 2008). All the images are 32 × 32 pixels size with three color channels (RGB).

This dataset is quite challenging, since it contains many variations of objects with natural backgrounds, in low resolution. Hence in order to tackle this dataset, algorithms must be able to find relevant information in noisy data.

**Figure 5:**
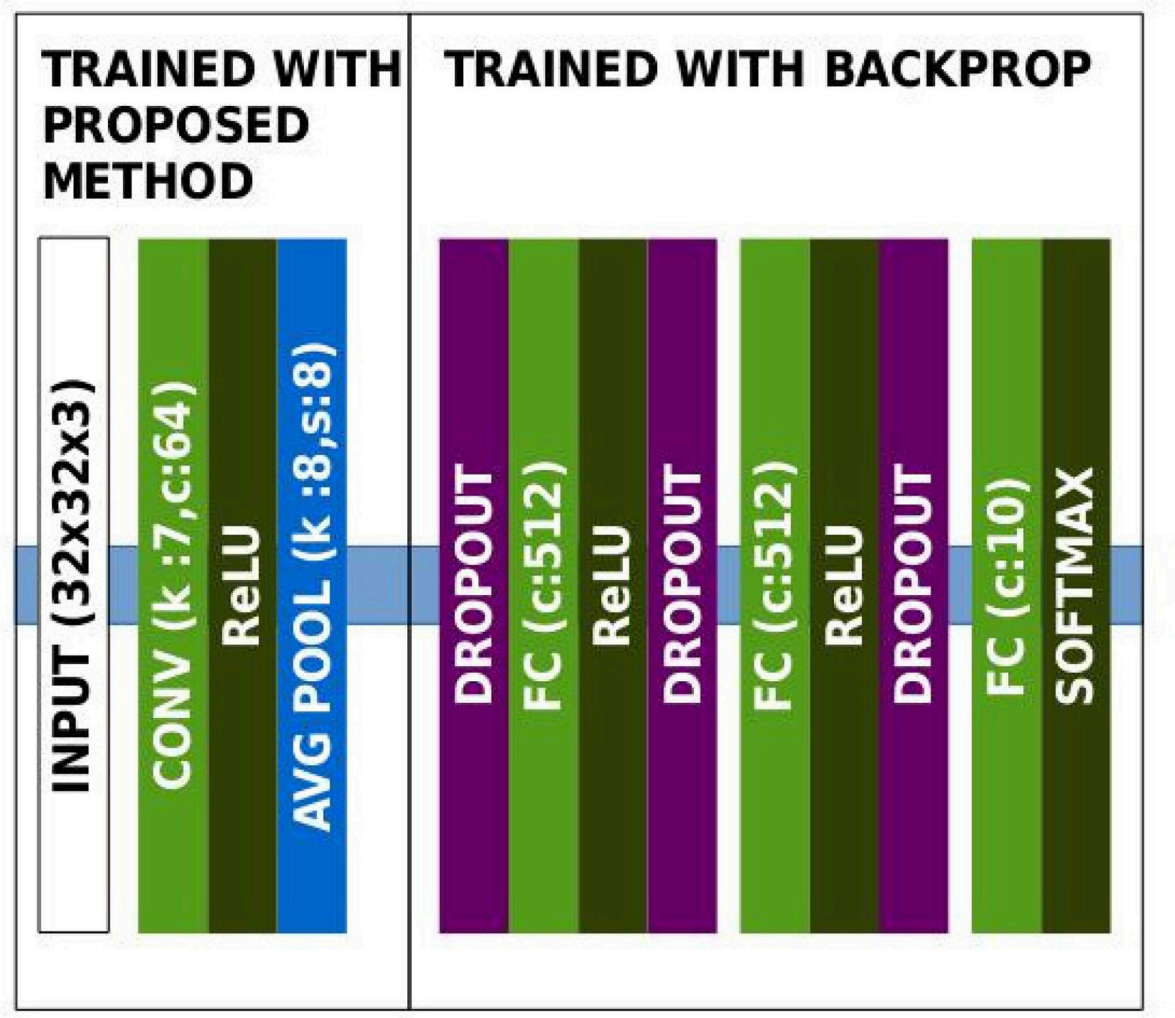
Architecture of the network in the CIFAR-10 experiment.

**Figure 6:**
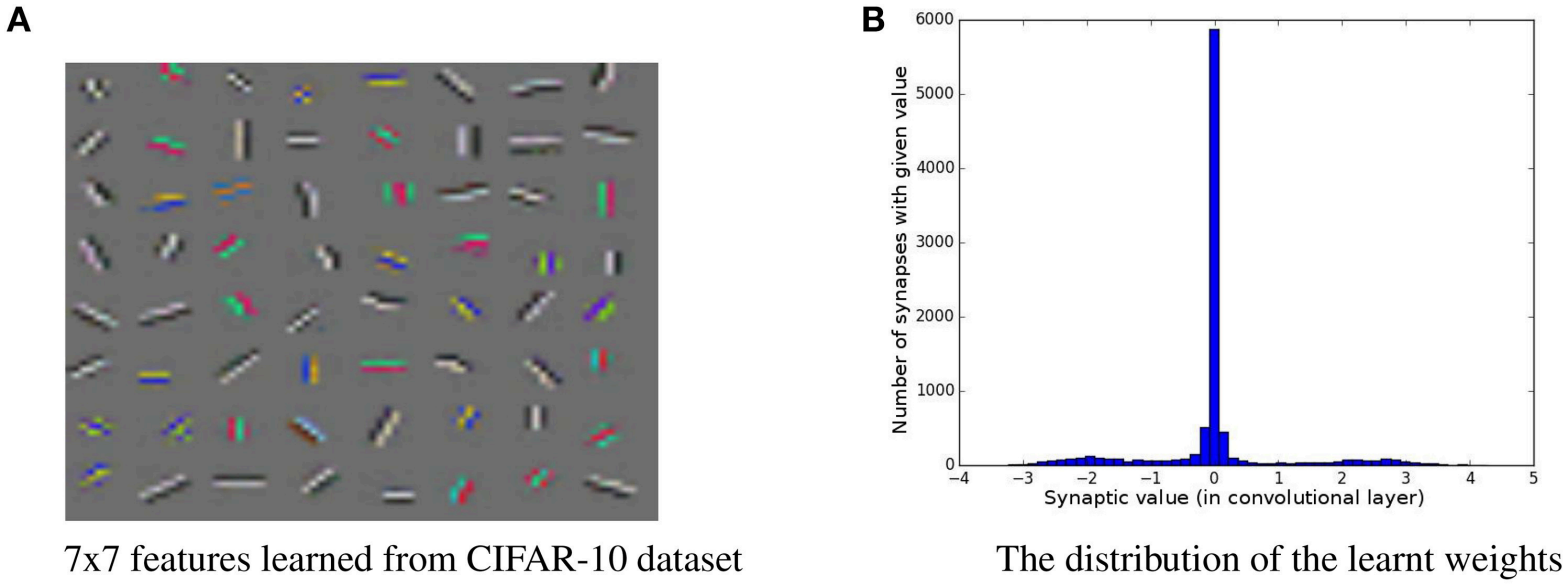
**(A)** Sixty-four filters of size 7 × 7 learned with our method on the CIFAR-10 dataset. **(B)** The weights distribution of the network's first layer trained on CIFAR-10.

The architecture used for this dataset is given in Figure 5. Learnt features are shown in Figure 6A. We observe that the features are similar to oriented-gabor features, which is consistent with the results of other unsupervised methods such as *k*-means and RBM. Also the weights distribution displayed in Figure 6B contains a majority of values close to zero, showing the sparsity of the features. Performances obtained on CIFAR-10, along with other methods evaluation, are shown in Table 4.

**Table 4:** CIFAR-10 results.

**Method**	**Unsupervised**	**Training samples**	**Accuracy (%)**
Triangle k-means (1,600 features) (Coates et al., 2011)	Yes	50,000	79.6
Triangle k-means (100 features) (Coates et al., 2011)	Yes	50,000	55.5
PCA-Net (Chan et al., 2014)	Yes	50,000	78.67
LIF CNN (Hunsberger and Eliasmith, 2015)	No	50,000	82.95
Regenerative Learning (Panda and Roy, 2016)	Yes	20,000	70.6
Our method (64 features)	Yes	5,000	71.2
CNN random frozen filters	No	50,000	55.3

As a performance baseline, we also trained the MLP with the same architecture but keeping the convolutional layer's weights randomly initialized and frozen. The increase of 17% of classification rate proves the usefulness of the features learnt with our method in the classification process.

Only a few works related to SNNs have been benchmarked on CIFAR-10. Cao et al. (2015) and Hunsberger and Eliasmith (2015) rely on convolutional to SNN conversion to perform supervised learning on the dataset. Panda and Roy (2016) built a convolutional feature hierarchy on the principle of auto-encoders with SNNs, and classified the top level representations with an MLP.

Also, some works unrelated to SNNs are worth comparing here. Coates et al. (2011) benchmarked four unsupervised feature learning methods (k-means, triangle k-means, RBM, and sparse auto-encoders) with only one layer. Results from the PCA-Net approach are also included.

Our approach reached good performance given the lightweight architectures and the limited number of samples. It outperforms the CNN with 64 random filters, confirming the relevance of the learnt features for classification, and also the Triangle K-means approach with 100 features. Empirically however, training with more samples without increasing the number of features does not improve the performance.

Also, due to the low resolution of CIFAR-10 images, we tried to add a second convolutional layer. The learnt filters in this new layer were very redundant and led to the same performance observed with only one layer. Further investigations might explore ways to force layers above the first to learn more sparse features.

### 4.5. STL-10

STL-10 is a dataset dedicated to unsupervised feature learning. Images were taken from the ImageNet dataset. The training set contains 5,000 images labeled over the same ten classes as CIFAR-10. An unlabeled training set of 100,000 images is also provided. Unlabeled images may contain objects from other classes of ImageNet (like bear, monkeys, trains…). The testing set contains 8,000 images (800 per class). All images are in RGB format with a resolution of 96 × 96.

We applied the same architecture as for the CIFAR-10 dataset, except the average pooling layer was done over 24 × 24 sized windows (in order to have the same 4 × 4 output dimension). As before, we limited the number of samples during the unsupervised learning step to 5,000.

While some works related to SNNs or STDP have been benchmarked on CIFAR-10, we were not able to find any using the STL-10 dataset. Hence our approach may be the first biologically inspired method trying to tackle this dataset.

Our approach reaches 60.1% accuracy on STL-10, which is above the lower-bound performance on this dataset. Performances obtained by other unsupervised methods range between 58 and 74%.

## 5. Discussion

The proposed approach is able to train lightweight convolutional architectures based on LIF neurons which can be used as a feature extractor prior to a supervised classification method. These networks achieve average levels of performance on four image classification datasets. While the performances are not as impressive as the ones obtained with fully supervised learning methods, where features are learnt specifically for the classification task, interesting characteristics emerge from this model.

By showing the equivalence between rank-order LIF neurons and perceptrons with ReLU activation, we were able to borrow computationally efficient concepts from both neuroscience and machine learning literature while remaining biologically plausible enough to allow the conversion of network trained this way to be converted into SNN.

Binary STDP along with WTA and synaptic normalization reduces drastically the process of parameters tuning compared to other STDP approaches. LIF neurons require the tuning of their respective time constant. STDP also requires four parameters to be tuned : the time constants $T_{+}$ and $T_{-}$ as well as the LTP and LTD factors *A*_+_ and *A*_−_ for each layer. Our model of binary STDP on the other hand only needs to set its learning rate λ, set globally for the whole architecture.

Another advantage over other STDP approaches is the ability to train the network with multiple images in parallel. While this ability is biologically implausible, it can become handy in order to accelerate the training phase thanks to the intrinsic parallel optimization provided by GPU. Also, the equivalence between LIF neurons and perceptrons with ReLU activation in presence of images allows us to perform the full propagation phase of a SNN in one shot, and to apply our STDP rule without the need of interpolation precise timing information from the image. Other approaches using SNNs with STDP requires the interpolation of temporal information from the image (Masquelier and Thorpe, 2007; Kheradpisheh et al., 2016), with gabor filters for instance, in order to generate spike trains. This way, STDP can be applied to learn the correlations between spike timings.

From a deep learning point of view, the main interest of our model resides in the proposal of a backpropagation-free training procedure for the first layers. As the backward pass in deep neural networks implies computationally heavy deconvolutions to compute the gradients of the parameters, any prior on visual modelization which can avoid a backpropagation over the whole network may help to reduce the computational overhead of this step. The LIF-ReLU equivalence demonstrated allows a convolutional network to take advantage of the inherent characteristic of STDP to quickly find repeating pattern in an input signal (Masquelier and Thorpe, 2007; Masquelier et al., 2009; Nessler et al., 2009).

With the WTA scheme proposed, we made the assumption that relevant visual information resides in the most contrasted patches. It also imposes the neurons to learn a sparse code with the combination of neighburhood and channel-wise inhibition. Such hard-coded WTA led to first layers features very similar to the gabor-like receptive-fields of LGN and V1. Quantitatively, the performances obtained on classification tasks allows us to conclude on the relevance of this learning process on such task. However it is still far from optimality considering the supervised learning methods (Graham, 2014; Hunsberger and Eliasmith, 2015) and human-level performances. The main drawback of our method is the difficulty to train more than one or two convolutional layers with. Since spatial inhibitions are critical in our WTA scheme to achieve feature sparseness, we suspect that the input width and height of one layer must be large enough to make the competition between neurons effective. Other competition schemes less dependent on the spatial dimension have to be explored in order to train deeper architectures with the proposed framework.

Also our binary variant of STDP rule shows the ability to train neurons with very low precision updates. Gradients used to be coded on floating-point variables ranging from 32 bits as these encoding schemes had the better trade-off between numerical precision and efficiency on CPU and GPU hardware. Gupta et al. (2015) showed the possibility to perform gradient descent with only 16-bits floating-point resolution, a feature implemented since then in NVidia Pascal and AMD RX Vega GPUs. Studies on gradient quantization (Zhou et al., 2016; Deng et al., 2017) showed promising results reducing the precision down to 2 bits without penalizing significantly the performances. The main advantage of such reduction in resolution is two-fold : the lowest the resolution, the fastest the computations (under the condition hardware has sufficient dedicated compute units) and the fastest the memory transfers. Seide et al. (2014) accelerated learning speed by a factor 50 quantizing the weight updates gradients on 1 bit, enabling a very fast transfer between the 8 GPU of the considered cluster. The binary STDP learning rule proposed here may fit this goal. Further quantization on activations and weights (even if the distributions obtained on MNIST and CIFAR-10 seem to converge to three modes) are to be studied in such framework in order to bring massive acceleration thanks to this biologically inspired method.

In order to better understand the implication of the binary STDP learning rule from a machine learning point of view, studies on the equivalence to state-of-the art methods should be performed as in Hyvärinen et al. (2004) and Carlson et al. (2013). Further mathematical analysis may help us understanding better the limits and potentials of our approach in order to combine it with other approaches. The literature in machine learning and neuroscience (accurately summarized in Marblestone et al., 2016) shows that it is unlikely that only one objective function or algorithm may be responsible for all the learning capabilities of the brain. Considered combinations include supervised approach with backpropagation compatible models such as Esser et al. (2015), reinforcement learning methods (Mnih et al., 2013; Mozafari et al., 2017), as well as other unsupervised strategies such as auto-encoders and GANs.

Finally, the binary STDP along with WTA and normalization has been shown to be successful at learning in an unsupervised manner low level visual features from image data. Extension of this learning framework on temporal data is envisaged. The roles of neural oscillations in the brain are still studied, and their place in attention-demanding tasks (Dugué et al., 2015; McLelland and VanRullen, 2016) is still under debate. Nevertheless, oscillation processes like the theta-gamma model (McLelland and VanRullen, 2016) shows interesting information segmentation abilities, and may be incorporated in a network of spiking or recurrent artificial neurons (such as GRU and LTSM) as a more hard-coded WTA scheme to evaluate their impact during learning.

## Author contributions

PF, FM, and ST: Designed the study; PF and FM: Analyzed the data; PF: Wrote the manuscript; PF, FM, and ST: Revised the manuscript, approved the final version, and agreed to be accountable for all aspects of the work.

### Conflict of interest statement

The authors declare that the research was conducted in the absence of any commercial or financial relationships that could be construed as a potential conflict of interest.
